# Expression of tricellular tight junction proteins and the paracellular macromolecule barrier are recovered in remission of ulcerative colitis

**DOI:** 10.1186/s12876-021-01723-7

**Published:** 2021-03-31

**Authors:** Jia-Chen E. Hu, Franziska Weiß, Christian Bojarski, Federica Branchi, Jörg-Dieter Schulzke, Michael Fromm, Susanne M. Krug

**Affiliations:** 1 Clinical Physiology / Nutritional Medicine, Charité - Universitätsmedizin Berlin, Campus Benjamin Franklin, Berlin, Germany; 2 Department of Gastroenterology, Rheumatology and Infectious Diseases, Charité - Universitätsmedizin Berlin, Campus Benjamin Franklin, Berlin, Germany

**Keywords:** Tricellulin, Ulcerative colitis, Tight junction, Macromolecule passage

## Abstract

**Background:**

Ulcerative colitis (UC) has a relapsing and remitting pattern, wherein the underlying mechanisms of the relapse might involve an enhanced uptake of luminal antigens which stimulate the immune response. The tricellular tight junction protein, tricellulin, takes charge of preventing paracellular passage of macromolecules. It is characterized by downregulated expression in active UC and its correct localization is regulated by angulins. We thus analyzed the tricellulin and angulin expression as well as intestinal barrier function and aimed to determine the role of tricellulin in the mechanisms of relapse.

**Methods:**

Colon biopsies were collected from controls and UC patients who underwent colonoscopy at the central endoscopy department of Campus Benjamin Franklin, Charité - Universitätsmedizin Berlin. Remission of UC was defined basing on the clinical appearance and a normal Mayo endoscopic subscore. Intestinal barrier function was evaluated by electrophysiological and paracellular flux measurements on biopsies mounted in Ussing chambers.

**Results:**

The downregulated tricellulin expression in active UC was recovered in remission UC to control values. Likewise, angulins were in remission UC at the same levels as in controls. Also, the epithelial resistance which was decreased in active UC was restored in remission to the same range as in controls, along with the unaltered paracellular permeabilities for fluorescein and FITC-dextran 4 kDa.

**Conclusions:**

In remission of UC, tricellulin expression level as well as intestinal barrier functions were restored to normal, after they were impaired in active UC. This points toward a re-sealing of the impaired tricellular paracellular pathway and abated uptake of antigens to normal rates in remission of UC.

## Background

Ulcerative colitis (UC) is one type of inflammatory bowel disease (IBD) first reported in late 1800s as idiopathic colitis with superficial ulcers of the colon and discharge of blood and mucus [1]. These days UC is recognized as continuous colonic inflammation starting from the rectum and spreading to proximal direction, with a natural course of relapsing and remitting and a common symptom of bloody diarrhea. Due to the climbing prevalence in western countries and developing incidence in developing countries, UC poses a heavy burden to worldwide health systems [2, 3].

In spite of recognition of several risk factors (i.e., genetic predisposition [4–6], environmental factors [7–9], and microbiota [10–12]), the exact pathogenesis of UC is not fully elucidated. Among all the other factors, the intestinal epithelial barrier—as both target and potential initiation factor of the disease—is of large importance. Anatomically, the intestinal epithelial barrier sits at the interface of the inner and outer environment of the body, which endows its functions on one hand of digestion and absorption and on the other hand of prevention of unwanted uptake of antigens and toxic substrates from the intestinal lumen. For preventing the latter, an intact and fully functional barrier is indubitably needed.

The tight junction (TJ) is a main component of the intestinal barrier which holds the most apical position between epithelial cells. The structure of the TJ is composed of the bicellular TJ (bTJ) which forms a belt-like meshwork between two epithelial cells [13] and the tricellular TJ (tTJ), a site where the converged bTJs strands vertically extend to the basal direction [14]. The tTJ has been considered to be a weak point of the epithelial barrier [15, 16]. Proteins of TJ are grouped into four families: (1) claudin, forming a family comprising 27 members in mammals [17], (2) junctional adhesion molecules (JAM) [18], (3) TJ-associated MARVEL (myelin and lymphocyte and related proteins for vesicle trafficking and membrane link) proteins (TAMPs) comprising occludin [19], tricellulin [15], and MARVEL D3 [20, 21], and (4) the angulin family containing three members, angulin-1 to -3 [22]. Among these, tricellulin and the angulin family are the two major components of the tTJ, both of which play a direct or indirect role in barrier function. In addition, angulins are responsible for the correct localization of tricellulin by recruiting it to tTJs [22].

In UC, observed changes of the TJ included downregulation of tricellulin and claudin-4, and upregulation of claudin-2 [23–25]. The reduced tricellulin could cause increased paracellular passage of macromolecules resulting in an enhanced antigen uptake while the increased claudin-2 often leads to increased paracellular passage of water and small solutes causing typical leak flux diarrhea. Although localized predominantly at the tTJ, tricellulin is essential for the maintenance of the whole TJ structure and barrier function, as knockdown of tricellulin was shown to cause morphological disorganization of both, bTJs and tTJs, and functional alteration like decreased transepithelial resistance (TER) and increased flux of 4 kDa fluorescein isothiocyanate-dextran (FD4) [15]. Overexpression of tricellulin exclusively at the tTJ led to unchanged permeability for small ions but extensive reduction of permeability for macromolecules up to 20 kDa, indicating the important role of tricellulin in preventing the passage of macromolecules [26].

In a recent study on intestinal biopsies from active UC patients, tricellulin was downregulated and the intestinal barrier was impaired, as evidenced by decreased epithelial resistance, which included increased subepithelial resistance due to the inflammatory processes and strongly decreased epithelial resistance reflecting the barrier impairment. Furthermore, increased permeabilities for fluorescein and FD4 underlined the barrier defects [25]. Therein, the relation between reduced tricellulin expression and impaired barrier function is of great interest and recalls the classical chicken-and-egg question, whether this downregulation of tricellulin causes increased luminal antigen uptake and subsequently initiates the immune response and defects the intestinal barrier or the other way around is the consequence of the impaired barrier. Exploration of this question might provide an insight in the pathogenesis of the disease and also would help to predict the probability of relapse.

In addition, in the other major type of IBD, Crohn’s disease (CD), it has been shown that the tricellulin expression level was unchanged, but its localization was shifted from crypts to surface epithelium [25]. One of the regulators of tricellulin localization, angulin-1, was downregulated in CD and by this was able to vulnerate the barrier function [27]. Therefore, angulins are also factors that could play a role during remission and relapse of IBD.

For this purpose, we collected colon biopsies from UC patients in remission and in active state, as well as from controls and aimed to explore the relation between tTJ proteins and intestinal barrier function by expressional analysis along with electrophysiological and flux measurements.

## Materials and methods

### Collection of colon biopsies and patient features

14 UC patients (4 active and 10 in remission) as defined by Ungaro et al. [28] and 24 patients who had colonoscopy and whose diagnosis for intestinal diseases were ruled out were enrolled for the study between 2017 and 2020 (Table 1). This study was approved by the local ethics committee (Ethics Committee of Charité - Universitätsmedizin Berlin, no. EA4/015/13), and all methods were performed in accordance with the respective approval. Patients were informed one day in advance and gave written consent to sampling additional biopsies to ones that were taken for diagnostics during the routine endoscopic examination. For these, patients allowed usage for analysis of electrophysiological, functional properties and of RNA and protein expression.

**Table 1:** Overview of characteristics of the enrolled patients

Characteristic	Control (n = 24)		UC_act_ (n = 4)	UC_rem_ (n = 10)
Age (median, range)	56 (24–66)		50 (35–60)	50 (35–60)
Gender (male/female)	8/16		3/1	2/8
Mayo Endoscopic Subscore (median)	–		5,5	0

From the 14 UC patients depending on the involved region, difficulty of the colonoscopy and other conditions occurred on the examination day, one to three pieces of biopsies from each patient were collected, as regional conditions might matter. A total of 36 pieces of biopsies from colon were collected.

The Mayo endoscopic subscore is the part within the full Mayo score to access disease presentation during colonoscopy [29]. Based on the aim of this study, patients with mild and moderate disease activity were unified as active, and remission of UC was defined based on the clinical appearance and a normal Mayo endoscopic subscore (Table 2).

**Table 2:** Detailed characteristics of the enrolled UC patients

UC Patient	Age	Gender	Mayo endoscopic subscore	Medication	Activity	Duration of remission
1	55	F	5	5-ASA	Mild	–
2	51	M	3	5-ASA	Mild	–
3	50	M	6	Unknown	Moderate	–
4	48	M	6	Unknown	Moderate	–
5	60	F	0	5-ASA	Remission	1 year
6	41	F	0	AZA	Remission	Unknown
7	50	F	0	Unknown	Remission	6 years
8	50	F	0	5-ASA	Remission	Unknown
9	35	F	1	5-ASA	Remission	3 years
10	49	M	0	5-ASA	Remission	1 year
11	53	F	0	5-ASA	Remission	Unknown
12	35	F	0	Unknown	Remission	9 years
13	59	M	0	Unknown	Remission	10 years
14	46	F	0	5-ASA	Remission	3 year

F: female, M: male

### Isolation of RNA and protein from colon biopsies

RNA and protein were isolated in parallel from the same sample using NucleoSpin RNA/Protein kit (Macherey–Nagel, Düren, Germany) according to the instruction of the manufacturer. The isolated RNA was quantified by NanoDrop ND-1000 UV–Vis Spectrophotometer (peqLab Biotechnologie GmbH, Erlangen, Germany). The concentration of protein was determined using bicinchoninic acid (BCA) protein assay.

### Reverse transcription and quantitative real-time polymerase chain reaction (qRT-PCR)

Reverse transcription was performed using High Capacity cDNA Reverse Transcription kit (Thermo Fisher, Mannheim, Germany). TaqMan® probes tricellulin (Hs00930631_m1), angulin-1 (Hs01076323_m1), angulin-2 (Hs01111433_m1), angulin-3 (Hs01025498_m1), Cldn-2 (Hs00252666_s1), Cldn-4 (Hs00533616_s1), and GAPDH (Hs02786624_g1) were used in qRT-PCR. A relative method was applied for expression analysis according to the 2^−ΔΔCT^ method.

### Western blotting

Western blotting was performed as previously described [27]. 20 μg of the obtained protein were electrophoresed using 12.5% sodium dodecyl sulphate–polyacrylamide gel and then transferred to a PVDF membrane (Perkin Elmer, Rodgau, Germany). After blocking with 1% polyvinylpyrrolidone-40 and 0.05% Tween-20, corresponding protein was detected by primary antibodies against tricellulin (1:2000, Invitrogen), angulin-1 (1:3000, Sigma), claudin-4 (1:1000, Invitrogen) and β-actin (1:10,000, Invitrogen) and secondary peroxidase-conjugated antibodies (anti-mouse or anti-rabbit, Jackson ImmunoResearch, Ely, UK). For detection, membranes were incubated with SuperSignal West Pico Plus Stable Peroxide solution (Thermo Fisher, MA, USA) and exposed in Fusion FX7 (Vilber Lourmat, Eberhardzell, Germany). Densitometric analysis was performed using Multi Gauge V2.3 software (FujiFilm, Japan). The protein expression was quantified after normalizing the respective band intensities by β-actin.

### Immunofluorescent staining

Formalin-fixed, paraffin-embedded sections were used to check the tricellular localization of tricellulin. For deparaffinization sections were washed 3× times with xylene and rehydration was achieved by washes in 100%, 90%, 80%, 70% ethanol and deionized water. For antigen recovery, slides were boiled in TEC buffer (2 mM Tris, 1,3 mM EDTA, 1 mM Tri-sodiumcitrat, pH 7.8) for 30 min using a microwave (at 360 W). Cooling down for 30 min was followed by blocking for 30 min in 4% goat serum in PBS and 30 min dako-blocking (Dako North America Inc. S3022). Samples were first incubated at 4 °C over night with primary antibodies (each 1:200, mouse anti-ZO-1 (Invitrogen and rabbit anti-tricellulin (abcam ab203567)) and afterwards for 90 min at room temperature in secondary antibodies (each 1:500, Alexa fluor 594 goat anti-mouse and Alexa Fluor 488 goat anti-rabbit, Molecular Probes MoBiTec) and DAPI (1:1000). Images were obtained using a confocal laser-scanning microscope with excitation wavelengths of 543 nm and 488 nm.

### Electrophysiological and flux measurements

Electrophysiological features were measured in Ussing chambers as described before [25]. Briefly, one-path impedance spectroscopy was applied to determine the electrical resistance of the whole tissue (R^t^, equivalent to TER), the resistance of the epithelial cell sheet (R^epi^), and the resistance of the subepithelial cell layers (R^sub^). Since R^epi^ and R^sub^ are resistors in series, their values add up to R^t^.

For measuring FD4 fluxes, 0.4 mM of pre-dialyzed FD4 (TdB Consultancy, Sweden) in apical hemi-chamber and 0.4 mM unlabeled dextran 4 kDa (Serva, Heidelberg, Germany) in basolateral side were applied. The measurements were carried out at 520 nm using a spectrometer (Tecan Infinite M200, Tecan, Switzerland) on basolateral samples taken at 0 min, 30 min, 60 min, 90 min, and 120 min after addition from the unlabeled side.

### Statistical analysis

All data are expressed as mean values ± standard error of the mean (SEM), indicating n as the number of single independent measurements. Statistical analyses were performed using Student’s *t*-test for comparison of two groups, or one-way ANOVA for comparing more than two groups (multiple testing) and *p* < 0.05 was considered significant (*p < 0.05, **p < 0.01, ***p < 0.001).

## Results

### Protein expression of TJ molecules in UC

**Fig. 1:**
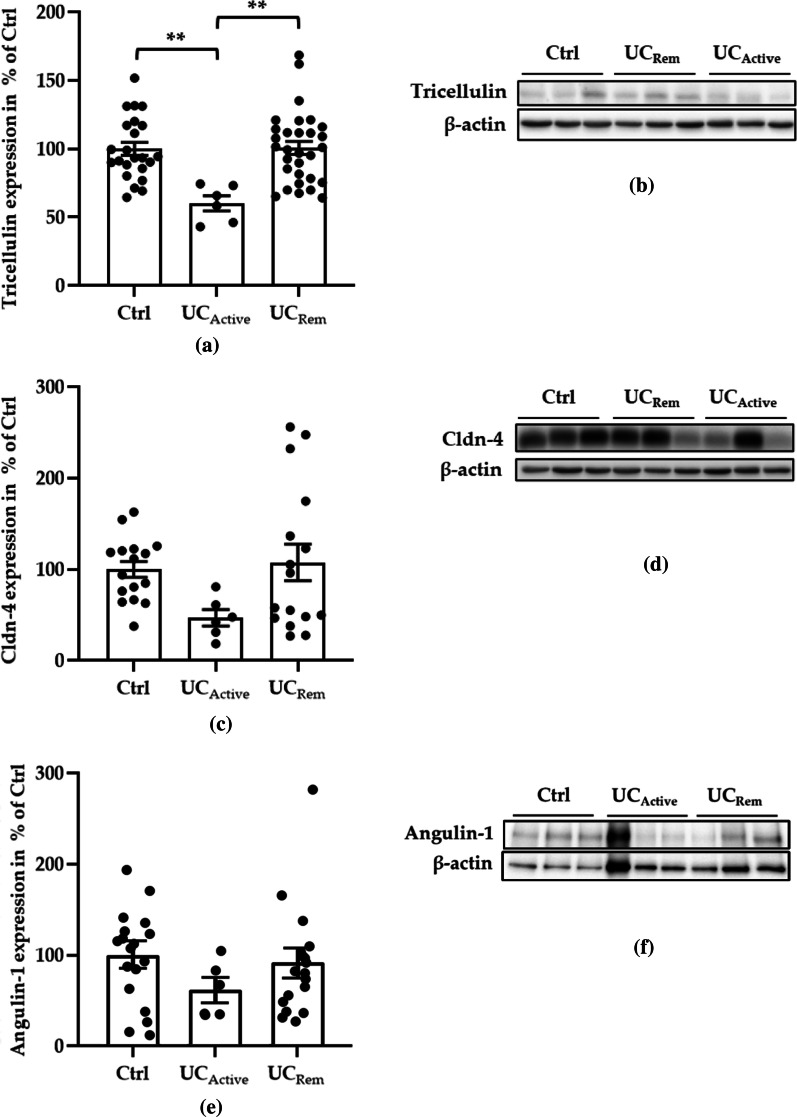
Tricellulin protein expression of human intestinal tissues. Mean value of Ctrl was set to 100%. **a** Combined scatter and bar plot of tricellulin of Ctrl, active UC (UC_Active_), and remission UC (UC_Rem_). In active patients, tricellulin was downregulated compared to Ctrl. Importantly, in remission of UC, the expression of tricellulin returned to the level of Ctrl (Ctrl: 100 ± 4.75%, n = 23; UC_Active_: 60.22 ± 5.47%; UC_Rem_: 100.69 ± 4.79%, n = 30, n = 6, **p < 0.01). **b** Representative western blots of tricellulin in Ctrl, UC_Rem_ and UC_Active_. **c** Scatter and bar plot of Cldn-4 of Ctrl (100 ± 8.70%, n = 16), UC_Active_ (46.89 ± 9.03%, n = 6), and UC_Rem_ (107.63 ± 20.06%, n = 16). **d** Representative western blots of Cldn-4 in Ctrl, UC_Rem_, and UC_Active_. **e** Scatter and bar plot of angulin-1 of Ctrl (100 ± 12.24%, n = 18), UC_Active_ (61.45 ± 12.36%, n = 6, *p* = 0.2008) and UC_Rem_ (92.04 ± 14.49%, n = 18). **f** Representative western blots of angulin-1 from Ctrl, UC_Active_ and UC_Rem_

The protein expression of tricellulin, claudin-4 (Cldn-4) and angulin-1 were analyzed using colonic biopsies from controls (Ctrl) and UC patients. At protein expression level, tricellulin was downregulated in active UC (UC_Active_) compared with Ctrl (Fig. 1a, b, ** *p* < 0.01), which was in line with findings of previous research [25]. In remission of UC (UC_Rem_), the decreased expression of tricellulin was recovered to a similar range as in Ctrl (Fig. 1a, b).

Cldn-4 expression showed a tendency below significance of reduction in UC_Active_ fitting to the previous results [25], while its protein expression in UC_Rem_ was unchanged compared to Ctrl (Fig. 1c, d). The expression level of the other major component of the tTJ, angulin-1 which played a role in Crohn’s disease when tricellulin expression remained unaltered [27], was also unchanged versus Ctrl (Fig. 1e, f), although a slight tendency (not significant) for a decrease in expression was detectable in UC_Active_.

### mRNA expression of TJ molecules in UC

**Fig. 2:**
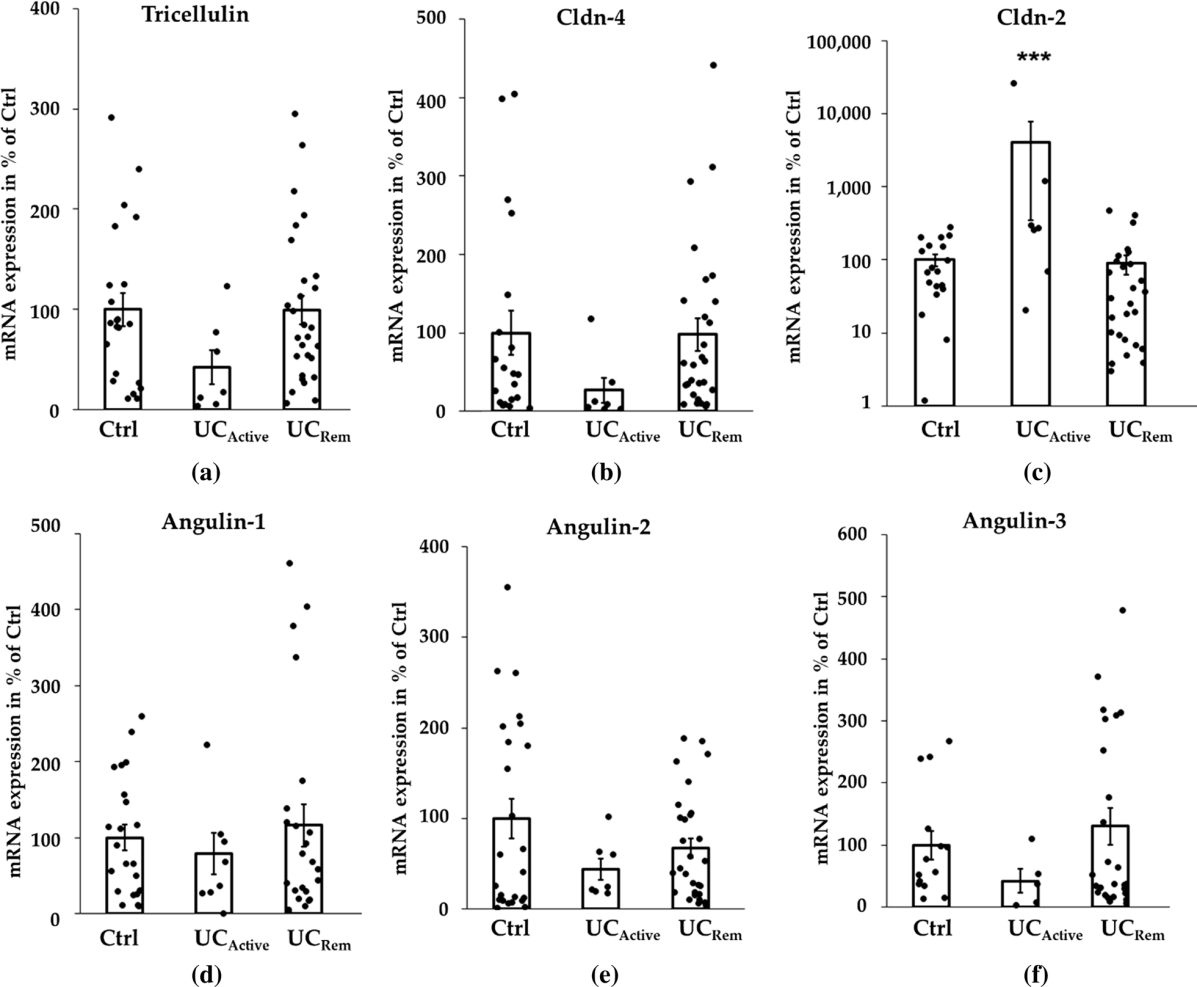
Combined scatter and bar plot of mRNA expression for TJ molecules. Mean value of Ctrl was set to 100%. **a** Tricellulin: Ctrl = 100 ± 16.75%, n = 22; UC_Active_: 42.51 ± 14.38%, n = 7, *p* = 0.1576; UC_Rem_: 99.25 ± 14.37%, n = 28, *p* = 0.9728. **b** Cldn-4: Ctrl = 100 ± 28.64%, n = 20; UC_Active_: 26.67 ± 15.86%, n = 7, *p* = 0.1540; UC_Rem_ = 97.58 ± 20.27%, n = 28, *p* = 0.9437. **c** Cldn-2: Ctrl = 100 ± 18.61%, n = 19; UC_Active_: 4065.38 ± 3714.70%, n = 7, ***p = 0.0006; UC_Rem_ = 89.66 ± 26.37%, n = 27, *p* = 0.748; for better visibility logarithmic scale was chosen for cldn-2. **d** Angulin-1: Ctrl = 100 ± 16.81%, n = 22; UC_Active_: 79.08 ± 27.60%, n = 8, *p* = 0.10496; UC_Rem_ = 116.13 ± 27.89%, n = 24, *p* = 0.6305. (**e**) Angulin-2: Ctrl = 100 ± 21.85%, n = 24; UC_Active_: 43.93 ± 12.14%, n = 7, *p* = 0.3728; UC_Rem_ = 67.14 ± 10.88%, n = 29, *p* = 0.1623. **e** Angulin-3: Ctrl = 100 ± 3.35%, n = 14; UC_Active_: 42.50 ± 19.34%, n = 5, *p* = 0.1814; UC_Rem_ = 130.56 ± 29.47%, n = 27, *p* = 0.4954

On mRNA expression level, no difference was found for tricellulin, Cldn-4, and angulin-1 in UC_Rem_ compared with Ctrl (Fig. 2a, b, d), which was in full accordance with the results of protein expression described above. We also investigated the mRNA expression of the other two members of the angulin family, angulin-2 and -3, as they also played roles in the barrier function [22] but currently we do not have reliable antibodies against them to acquire information at protein expression level. In UC_Rem_, the mRNA expression of angulin-2 and -3 was unchanged compared to Ctrl (Fig. 2e, f), although in UC_Active_ there seemed to be a tendency for downregulation as already observed for tricellulin or Cldn-4. It is also worth to be mentioned that angulin-3 was a low-abundant RNA that was not detectable in all samples, suggesting that it plays a less important role than the other two angulins in the intestine. Claudin-2 (Cldn-2) known to be highly upregulated in UC_Active_ [25], we were not able to detect on protein level due to the same reason of current antibody quality. However, we were able to investigate the mRNA expression level and found it strongly expressed in UC_Active_ and in very low amounts also in controls and during remission (Fig. 2c).

### Localization of tricellulin

**Fig. 3:**
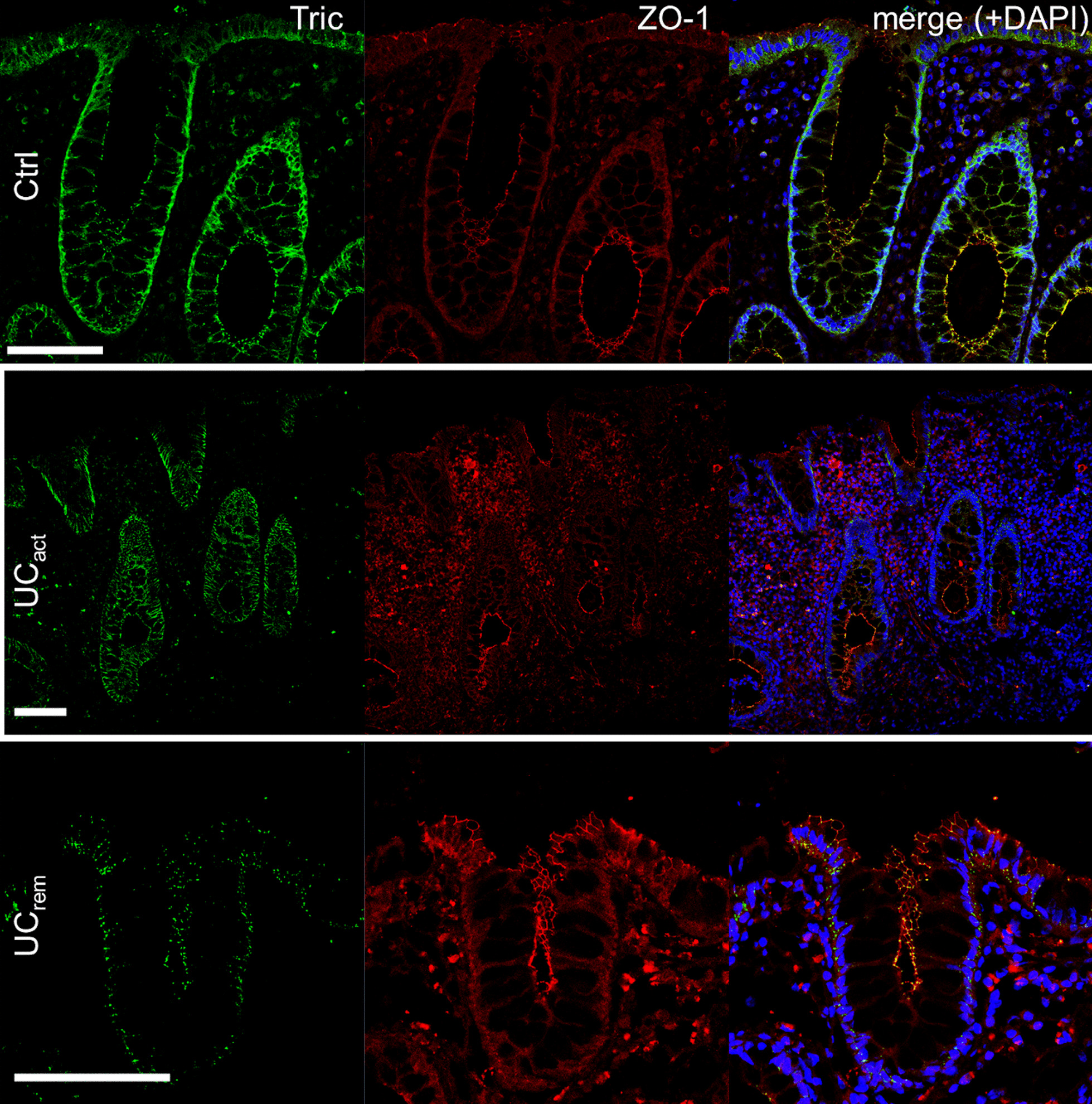
Representative immunofluoresecence staining for assessing localization of tricellulin (TRIC, green) and ZO-1 (red) within intestinal tissues. The signal intensities for tricellulin were increased in UC_Act_ images, also in background stainings. Tricellulin was localized at tricellular contacts (counterstaining ZO-1) and was evenly distributed along the crypts in all three groups. Bar = 100 µm

As the tricellular localization of tricellulin or its distribution within the intestinal epithelium might be affected even by small changes, we checked its localization within the three cohorts. Although it was clearly downregulated in active patients, no shift in localization was obvious, as also already shown in a former study [25]. Again, in patients in remission higher expression levels and tTJ localization was maintained (Fig. 3).

### Parameters of intestinal permeability

**Fig. 4:**
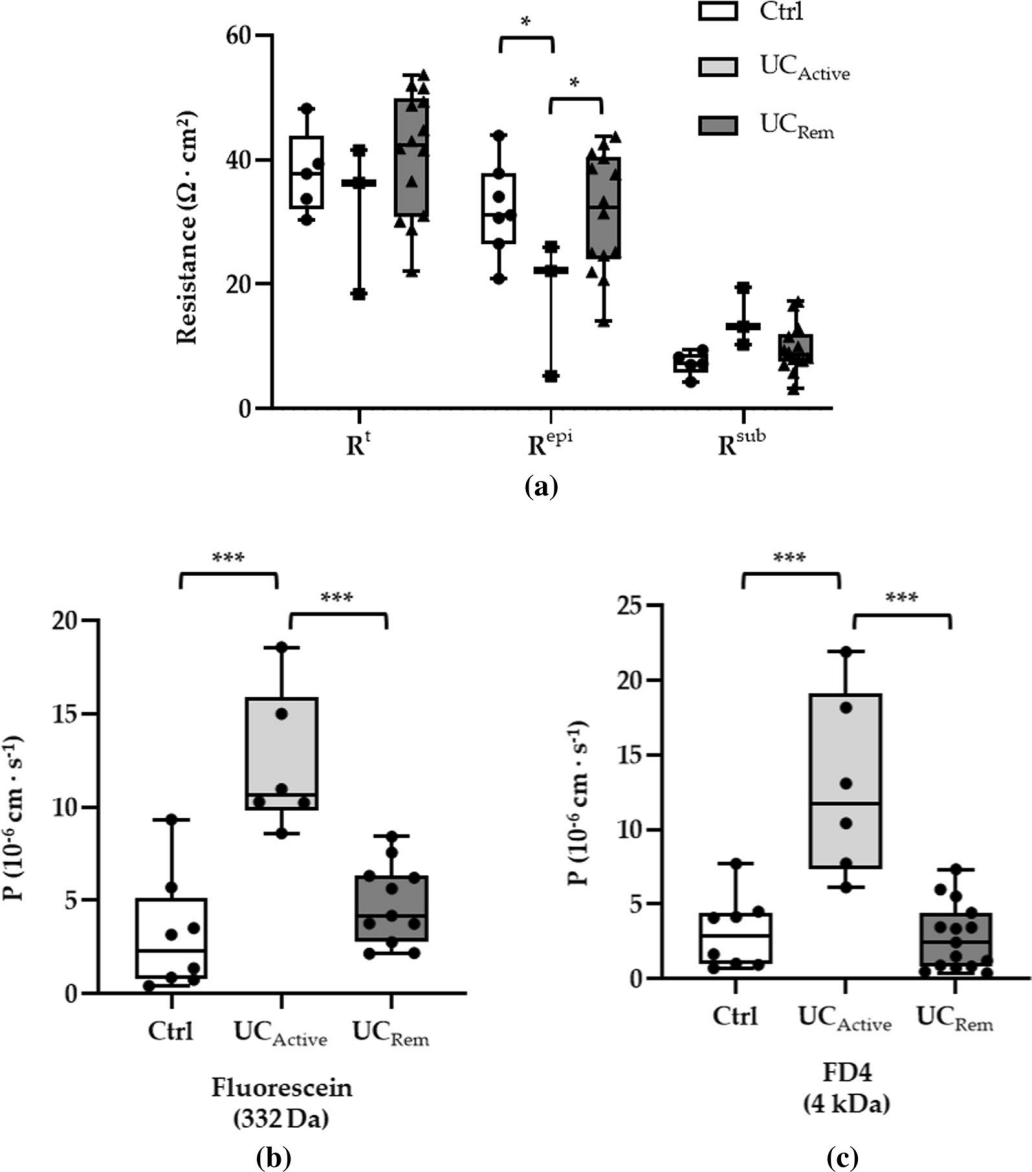
Parameters of permeability of intestinal biopsies in Ctrl, UC_Active_, and UC_Rem_. **a** Electrical resistances of colon biopsies of Ctrl, UC_Active_, and UC_Rem_. UC_Rem_ showed the same level of transepithelial resistance (R^t^ equivalent to TER), epithelial (R^epi^), and subepithelial resistance (R^sub^) as in Ctrl (Ctrl: R^t^ = 37.89 ± 3.02 Ω · cm^2^, R^epi^ = 30.62 ± 3.79 Ω · cm^2^, R^sub^ = 7.26 ± 0.85 Ω · cm^2^, n = 5; UC_Rem_: R_t_ = 41.08 ± 2.65 Ω · cm^2^, R^epi^ = 31.45 ± 2.53 Ω · cm^2^, R^sub^ = 9.63 ± 1.03 Ω · cm^2^, n = 14). UC_Active_ has lower R^epi^ (17.8 ± 2.53 Ω · cm^2^, n = 3) compared with Ctrl (*p < 0.05) and UC_Rem_ (*p < 0.05), together with an increasing, albeit not significant, tendency of R^sub^ (14.29 ± 2.69 Ω · cm^2^, n = 3) leading to the same level of R^t^ (32.09 ± 7.00 Ω · cm^2^, n = 3). **b** Permeability for fluorescein. In Ctrl and UC_Rem_, the permeability stayed at the same range, both of which are lower than in UC_Active_ (Ctrl: 3.15 ± 1.09 × 10^–6^ cm · s^−1^, n = 8; UC_Rem_: 4.82 ± 0.65 × 10^–6^ cm · s^−1^, n = 11; UC_Active_: 12.29 ± 1.53 × 10^–6^ cm · s^−1^, n = 6, ***p < 0.001). **c** Permeability for FD4. Similar results were observed that UC_Active_ had higher permeability than Ctrl and UC_Rem_ (Ctrl: 3.10 ± 0.87 × 10^–6^ cm · s^−1^, n = 8; UC_Rem_: 2.81 ± 0.57 × 10^–6^ cm · s^−1^, n = 15; UC_Active_: 12.91 ± 2.50 × 10^–6^ cm · s^−1^, n = 6, ***p < 0.001)

As for barrier function for ions, one-path impedance spectroscopy showed that common transepithelial resistance (R^t^ = TER) was not changed in UC_Rem_ compared with Ctrl (Fig. 4a). Importantly, the resistance of the epithelial layer (R^epi^)—which included the resistance of the TJ pathway—was reduced in UC_Active_ and restored to normal in UC_Rem_. The resistance of the subepithelial tissues (R^Sub^)—due to inflammatory alterations in UC [30]—behaves in the opposite direction (albeit not significant). As R^epi^ and R^Sub^ change in opposite direction they add up producing unchanged R^t^.

Permeability for fluorescein also remained unaltered in UC_Rem_ in comparison with Ctrl (Fig. 4b), which was in line with the previous finding of permeability for 400 Da FITC-sulfonic acid [31]. For macromolecule, permeability for FD4 was within a similar range in UC_Rem_ and Ctrl as well (Fig. 4c).

Regarding active UC, a reduced epithelial resistance and a tendency of raised subepithelial resistance (not significant) led to an overall unaltered transepithelial resistance compared with Ctrl (Fig. 4a). Elevated permeability for both fluorescein and FD4 was observed in comparison with Ctrl as well as UC_Rem_ (Fig. 4b, c, ***p < 0.001). These results in active UC were in line with previous findings by Krug et al. [25].

## Discussion

UC is a chronic inflammatory disease with a relapsing and remitting pattern, yet the mechanisms of its relapse are still not fully understood and need to be explored. Previous research found that tricellulin, a tTJ protein responsible for preventing macromolecule passage, was downregulated in active UC compared with control patients [25]. Furthermore, in exploration of intestinal barrier function, it was observed that the passage of tracers which were known to follow the paracellular pathway as fluorescein and FITC-dextran 4 kDa was also increased [25].

These results raised the classical chicken-and-egg question regarding the initiation of the inflammation in UC. During the active phase of the disease, there is no doubt that impaired tTJs represented by tricellulin downregulation result in increased passage of luminal antigens and exacerbation of the inflammation. However, the prior factor therein remains to be determined, whether downregulation of tricellulin is the reason or the consequence of the inflammation. In addition, it raised the question whether during remission the paracellular uptake of antigens due to potentially impaired tTJs is still ongoing and thus support the development of a relapse.

Therefore, in this study, we collected intestinal biopsies from controls, UC patients in remission, and for a direct comparison within our present study a few active UC samples and investigated expression levels of several TJ proteins. Biopsies from active UC patients were more seldom (n = 4) than those of the other two groups, however the results of the UC_Active_ group had been established already in a recent study of this lab [25].

As the main important finding of the present study tricellulin expression was found to be recovered towards normal in patients in complete remission. A similar tendency was also observed for tricellulin but still with a diminished claudin-4 expression when active and less active UC patients were analyzed [32]. In contrast to this study, our present analysis incorporated only patients without any inflammatory activity (with a normal Mayo endoscopic subscore), which enables us to conclude on a lack of any tricellulin impairment in complete remission. This is important to rule out a primary barrier defect, which would contribute as a barrier component to the etiology of UC.

In previous IBD research, also Cldn-4 was also found to be downregulated in active disease [24]. In our present group of only 4 patients, there was only a tendency towards a decrease, which did not reach statistical significance. However, this was due to the low number of biopsies. In UC in remission, however, Cldn-4 expression was clearly restored to normal.

Other TJ proteins analyzed, Cldn-2 and angulin-1 to -3, exhibited comparable levels as in controls. Unfortunately, all three Cldn-2 antibodies currently available in our lab did not recognize Cldn-2 from human biopsies, because neither did the antibodies identify signals from remission UC nor from active patients that served as well-known positive controls for Cldn-2 upregulation, thus only mRNA expression was analyzed. These clearly showed an upregulation of Cldn-2 in active patients. For angulins, the mRNA expressions of all three angulins were unaltered in remission patients. Since angulin-1 was shown to be the major member of the family in colon [22] and was downregulated in CD [27], we also investigated the protein expression level of angulin-1 and also found it unchanged in remission UC.

As functional read-outs, we measured the ion permeability and fluxes of paracellular markers in biopsies in specialized Ussing chambers. What we found was that in remission of UC, the intestinal barrier function was no longer impaired, represented by the comparable TER (consisting of epithelial and subepithelial resistance) as in controls, along with the similar paracellular fluxes of fluorescein and FD4. As control, we also analyzed samples from active patients. Akin to a previous study [25], decreased epithelial resistance as well as increased permeabilities for fluorescein and FD4 were observed, indicating an impaired barrier function in active UC. These findings demonstrate a complete functional restoration of the intestinal barrier during remission also occurring for the tTJ, which was not known so far and which is important, since the altered tTJ can be a major site for unwanted macromolecule and antigen uptake into the mucosa.

Although we could not confirm the hypothesis of an ongoing paracellular antigen uptake during remission that could lead later to relapse of UC, we cannot exclude that changes in the tTJ might happen shortly before relapse as shown for the general TJ barrier in Crohn’s disease by Wyatt and coworkers [33]. None of the analyzed patients had a relapse since investigation, therefore the exploration of the relation between tricellulin and macromolecules passage in the pathophysiology of UC might require further investigation at more precise time points, for example shortly before the relapse of the disease. This, however, is not predictable so far and needs long-term follow-up studies to acquire applicable samples. If then in such kind of study the initial hypothesis of reduced tricellulin or angulin expression would be observed, these could provide a mechanistic insight into the pathogenesis of UC. However, although we cannot rule out a tTJ defect immediately preceding the onset or an acute episode as part of the inflammatory process, our data clearly points against a primary barrier defect within the tTJ in UC.

## Conclusions

Proteins of the tTJ, especially tricellulin, regulate paracellular macromolecule passage and contribute to maintaining the overall TJ barrier function. In active UC, tricellulin was recently found to be downregulated and the intestinal barrier function was impaired [25], wherein the relation between these two factors awaited exploration, as an enhanced passage of luminal antigens might be a reason for relapse of UC. Here for the first time we demonstrate that molecular as well as functional parameters of the TJ and especially the tTJ change in parallel in UC_Active_ and then are restored to normal in UC_Rem_. However, elucidation of the mechanism of relapse demand for further investigation, preferably at the time point shortly before relapse to allow further understanding of this still not predictable event.

## Data Availability

The data analysed during this study are included in this published article. Original data files, e.g. recordings used for that are available from the corresponding author on reasonable request.

## Abbreviations

bTJ: Bicellular tight junction
CD: Crohn’s disease
Cldn: Claudin
FD4: FITC-dextran 4 kDa
FITC: Fluorescein isothiocyanate
IBD: Inflammatory bowel disease
MARVEL: Myelin and lymphocyte and related proteins for vesicle trafficking and membrane link
R^epi^: Electrical resistance of the epithelial cell sheet
R^sub^: Electrical resistance of the subepithelial cell layers
R^t^: Electrical resistance of the entire tissue (transepithelial resistance) R^t^ = R^epi^ + R^sub^
qRT-PCR: Quantitative real-time polymerase chain reaction
TAMP: TJ-associated MARVEL protein
TER: Transepithelial resistance, equivalent to R^t^
TJ: Tight junction
tTJ: Tricellular tight junction
UC: Ulcerative colitis
